# On the Chemico-Physiological Effects of Coffee

**Published:** 1854-04

**Authors:** Julius Lehman


On the Chemico-Physiological Effects of Coffee.—By Julius Lehman.—If we consider the results of all the investigations and experiments on this subject we must arrive at these conclusions:

1st. That the use of the decoction of coffee produces two important actions in the system, which are difficult to harmonize, viz: stimulating the vascular and nervous system to greater activity, and at the same time considerably retarding the metamorphosis.

2nd. That the stimulating effect above mentioned, so valuable to us from its reviving the wearied spirit, and disposing to thought and promoting a feeling of comfort and cheerfulness, is due to the opposing modifications of the action of empyreumatic oil and caffein.

3rd. That the retarding of the metamorphosis is mainly due to the oil—the caffein showing this, *only* when it exists in large quantities.

4th. That increased activity of the heart, trembling, dizziness, delirium, intoxication, &c., are the effects of caffein.

5th. That the increased action of the perspiratory organs, of the kidneys, of the peristaltic action, and of the brain, are the effects of the empyreumatic oil.

If the decoction be too strong, that is to say, if it is prepared in such a way as to contain too great a quantity of both substances for the system, then occur the effects peculiar to both, trembling, activity of the heart, congestion, &c., &c. If we consider these two chief actions of coffee, which are peculiar to some other substances, such as tea, cocoa, spirituous liquors, &c., though in a modified degree, we observe that they are at variance with the general law, that the greater the expenditure of mental and bodily activity, the greater the waste—or rather the metamorphosis of the system. Whether this excitement of the vascular nervous system, occasions more rapidly the destruction of the processes of life, or how these two opposing effects are to be explained, remains for the future to discover.

One who is familiar with the general law above mentioned, that an increase or decrease of mental and bodily expenditure, is attended by a proportionate increase ot decrease of the metamorphosis, will find it difficult to comprehend the strength, busy life and good health of the poorer classes, when he considers these in connection with the very small amount of actual nourishment that they are able to obtain. And, in fact, the existence of such persons would have been inconceivable, if no means of supporting their health and strength had been provided, besides that small amount of actual nourishment. Limited to that alone, there would have been a great disproportion between the amount received and that thrown off; the tissues must have suffered a continual waste, even to the dissolution of life. But instinct has taught them to make use of a substance, which is capable of making a too scanty nourishment sufficient to the system, and by preventing in this way the otherwise unavoidable disturbances of the balances of life.

It is particularly coffee, tea, cocoa, empyreumatic oils and alcoholic liquors, that possess this peculiar influence upon the system, and most of these agree in producing that excitement of the nervous system of so great importance in social life.

If we now reflect upon the distribution of these substances, we find that one of them is used by the people as a common article of food, while the others are enjoyed by the higher classes as articles of luxury. In those countries where one or other of these substances is cultivated, its culture is considered as a matter of chief importance by the people; thus, in Arabia, coffee; in China, tea; in the wine-producing countries, wine. On the contrary, where this is not the case, the choice has fallen almost exclusively on coffee or tea. The reason why the preference is given to these, particularly in Europe, is owing principally to the fact that they exert a far less injurious influence upon the system than alcoholic drinks, and also on account of their valuable action upon the nervous system from the united effects of coffee and empyreumatic oil. For while, by stimulating the reason and imagination, these prepare man for intellectual and bodily exertion; spirituous liquors exciting only his imagination, which degenerates by slight excess into confusion of thought; cause, by irritating the nervous system, general debility. These latter, therefore, can be used with much less safety than coffee or tea.

An attentive consideration of the quality and quantity of the composition of coffee and tea, the various modes of preparing them, as well as their action upon the system in connection with the kind of food used, will probably account for the peculiar preferences of nations, for the one over the other. If the composition of tea leaves be compared with roasted coffee, we find that they both possess those substances that are of such great importance—thein, etherial oil, and protein substance. The only difference between them is, that the coffee contains an aromatic substance, and the tea a greater amount of thein, but especially of etherial oil. Three most important effects are produced by these constituents:—1. The retarding of metamorphosis produced by the thein; but more particularly by the aromatic substance in coffee. 2. The lengthened activity of the brain, produced by the special action of thein and etherial oil. 3. Serving as an actual means of nourishment, by the amount of protein substance contained in them. Coffee influences more directly the metamorphosis, and tea the nervous system. In order to obtain completely these effects of the two drinks, they must be partaken of in substance. This occurs, however only among few nations, and is probably dependent upon the kind of food used, and their mode of life. The Orientals and Arabs regard coffee as a necessary article of food, and from their custom of drinking it with the dregs, thus making the large amount of protein substance and inorganic constituents serve as nourishment, have unconsciously rendered their very frugal diet less sensible to them. If the plastic ingredients are not of themselves sufficient, yet here comes to their assistance the indirect nourishing property of caffein and the aromatic substance—equalizing their necessities, and at the same time exciting their nervous system.

The Central Asiatic inhabitants of the Steppes, the Buratians, the Mongolians, &c., &c., make use of tea as a common article of food. They prepare it, first, by rubbing the leaves together, then boiling it in water, adding a little salt to it; after they have poured off the decoction from the dregs, they add to it butter and milk, and meal if they have any, which they roast before adding to the decoction. A person takes per day from twenty to forty cups. But even without meal, and only with a little milk, this tea often serves for weeks long, as the only means of nourishment. We see here again a people instinctively directed, as it were, in the peculiar mode of preparing tea; unconsciously adding those substances which are of such great importance, viz: the protein, that becoming soluble by boiling in salt water, and also a great portion of the inorganic constituents. It is probably here the large amount of thein that influences the metamorphosis. Of course they lose entirely the valuable property of the united action of etherial oil and thein, since by boiling, the former is dissipated. This could better be spared though than the other action of tea, which serves as a direct nourishment, which is of greater importance.

The mode of preparing these drinks among Europeans, is very different—not placing much value upon the nutritive substances contained in them, but merely upon those which are capable, indirectly, of nourishing, and those which induce greater activity of the nervous system and the brain. The mode of preparing coffee among the Germans, enables them to obtain, besides the empyreumatic oil, as much caffein as they can possibly obtain from it; while in the preparation of tea, less attention is paid to the amount of thein, than to the etherial oil—the whole amount of which passes over to the decoction. Hence, with them, tea acts only as a stimulus to their brain and nervous system, whilst coffee retards the metamorphosis, and, also, though not in so great a degree as tea, stimulates the nerves. The English, who produce so much meat in their own country, and can generally obtain so much of it that it is even possible for the poorer classes to partake of it daily, besides the protein substances, have less need to prepare their coffee in such a manner as to obtain from it indirect nourishment. Since, however, they feel the want of spirits and excitement, their choice has fallen upon tea, which promotes these better than coffee.

We find among the poorer classes of Germany, where meat is so rare, and considered so great a luxury, that they are obliged to substitute for it such poor food as potatoes, &c., which consist of substances which indeed satisfy their hunger, but yet are not fitted to prepare them for very great exertions. The feeling of mental and physical lethargy experienced by them, and produced by this spare diet, becomes much more evident if they suffer from the want of coffee. The rapid diffusion and yearly increase of the consumption of coffee, is an evidence of the great importance it has attained in social life.

Whilst, at the beginning of the last century, it was considered a luxury by the higher classes, it has now become a necessary article to all classes, wherever food is scarce and dear. Of its total production, which amounts annually to 600,000,000 of pounds two-thirds are consumed by the Europeans, whose exertions appear so disproportioned to the actual means of nourishment. In the Zollverein States of Germany, the consumption of it amounted, in 1851, to one hundred millions of pounds, or one-sixth of the total production. Upon each reduction of duty, millions of pounds more were consumed by the people. The consumption of coffee and potatoes, from the period of their general introduction, have gone hand in hand. We see the poor instinctively valuing coffee more, the more they are limited to potatoes as their chief food.

*The Substitute* (*Surrogate*) *for Coffee.*—At the time of the Continental embargo, the want of coffee was felt for the first time in Germany, and the necessity of possessing some such drink, led to the adoption of other substances produced there, to which, by roasting, a taste similar to that of coffee could be imparted. Notwithstanding the general introduction of coffee again, and its greatly increased production in the colonies, and the reduction of duties, which naturally made it cheaper, the consumption of the substitute was not lessened, but increased so as yearly to amount to 100,000 cwt. The opinion that Knapp, as well as other authors, has started, that this substitute is entirely deficient in those substances so valuable to the poorer classes, can be refuted if we examine its composition, and remove the idea that caffein is the only efficacious substance in coffee. The materials which they have chosen as substitutes, and which may be increased indefinitely, all undergo the same process as coffee, viz: roasting; by which the aromatic substance is evolved, and by which is effected that retardation of the metamorphoses of such great importance to the poorer classes. The roasting thus produces not only a taste similar to coffee, but also imparts to it one of its most important properties. Still we cannot be indifferent to the great deficiency that exists in the substitute; viz: the total absence of caffein, thus losing that valuable influence upon the vascular and nervous system and upon the brain. Since, however, surrogate is about from five to eight times cheaper than coffee, and possesses a part of the peculiar action of the latter, we cannot wonder at its having attained, among the poor, to such a general use.—*Med. Examiner.*

